# Long-term rise in riverine dissolved organic carbon concentration is predicted by electrolyte solubility theory

**DOI:** 10.1126/sciadv.ade3491

**Published:** 2023-01-18

**Authors:** Donald T. Monteith, Peter A. Henrys, Jakub Hruška, Heleen A. de Wit, Pavel Krám, Filip Moldan, Maximilian Posch, Antti Räike, John L. Stoddard, Ewan M. Shilland, M. Gloria Pereira, Chris D. Evans

**Affiliations:** ^1^ UK Centre for Ecology and Hydrology, Lancaster Environment Centre, Library Avenue, Bailrigg, Lancaster LA1 4AP, UK.; ^2^ Czech Geological Survey, Klárov 3, 11821 Prague, Czech Republic.; ^3^ Global Change Research Institute, Czech Academy of Sciences, Bělidla 986/4a, 603 00 Brno, Czech Republic.; ^4^ Norwegian Institute for Water Research (NIVA), Oslo, Norway.; ^5^ Centre for Biogeochemistry in the Anthropocene, Department of Biosciences, Section for Aquatic Biology and Toxicology, University of Oslo, Oslo, Norway.; ^6^ IVL Swedish Environmental Research Institute, Box 530 21, 400 14 Göteborg, Sweden.; ^7^ International Institute for Applied Systems Analysis (IIASA), A-2361 Laxenburg, Austria.; ^8^ Finnish Environment Institute (SYKE), P.O.Box 140, FI-00790 Helsinki, Finland.; ^9^ US EPA, Corvallis, OR 97333, USA.; ^10^ Environmental Change Research Centre, UCL, Gower Street, London WC1E 6BT, UK.; ^11^ Life Sciences, Natural History Museum, Cromwell Road, London, SW7 5BD, UK.; ^12^ School of Biological and Behavioural Sciences, Queen Mary University of London, Mile End Road, London E1 4NS, UK.; ^13^ UK Centre for Ecology and Hydrology, Environment Centre Wales, Deiniol Road, Bangor, LL57 2UW, UK.

## Abstract

The riverine dissolved organic carbon (DOC) flux is of similar magnitude to the terrestrial sink for atmospheric CO_2_, but the factors controlling it remain poorly determined and are largely absent from Earth system models (ESMs). Here, we show, for a range of European headwater catchments, that electrolyte solubility theory explains how declining precipitation ionic strength (IS) has increased the dissolution of thermally moderated pools of soluble soil organic matter (OM), while hydrological conditions govern the proportion of this OM entering the aquatic system. Solubility will continue to rise exponentially with declining IS until pollutant ion deposition fully flattens out under clean air policies. Future DOC export will increasingly depend on rates of warming and any directional changes to the intensity and seasonality of precipitation and marine ion deposition. Our findings provide a firm foundation for incorporating the processes dominating change in this component of the global carbon cycle in ESMs.

## Teaser

Teaser:

More dilute rainwater, due to less polluted air, dissolves more soil organic matter, resulting in the browning of rivers.

## INTRODUCTION

The global riverine carbon (C) flux, of circa 1.7 to 2.7 Pg year^−1^ (*1*), is similar in magnitude to C uptake by both the land surface and the oceans (*2*). In regions where wetter, and/or cooler, climates have led to the development of organic-rich soils, dissolved organic carbon (DOC) is often the dominant form of the exported C that also includes particulate and inorganic forms (*3*). DOC concentrations and fluxes in waters draining these systems are highly dynamic and have increased over much of northern Europe and northeastern America over the past three to four decades (*4*–*6*). These changes are important not only for the lateral export of C to the oceans per se but also for the ecological and biogeochemical structure and functioning of aquatic ecosystems because DOC provides a substrate for heterotrophic respiration (*7*) and limits aquatic primary productivity by attenuating photosynthetically active radiation (*8*). From a societal perspective, rising DOC concentrations in upland drinking water sources are a major concern for water companies with legal requirements to maintain predisinfection DOC concentrations at low levels and are thus faced with mounting water treatment costs (*9*) and uncertainty over future treatment infrastructure needs.

A range of process-based models have been developed to evaluate drivers of DOC variation and change in temperate (*10*), boreal (*11*), and Arctic (*12*) environments. While valuable for the development of scientific understanding, most are highly parameterized, require substantial site-specific calibration, and therefore have limited potential for upscaling. Although some have attempted to capture the processes determining longer-term DOC concentration change (*13*, *14*), this has arguably been addressed more effectively by statistically based attribution, in which rates of change in DOC have been linked to surrogates of acid deposition (*4*, *6*, *15*) and hydrological metrics such as discharge (*16*). Correlative model structures, however, can be challenging to interpret mechanistically. Ultimately, the appropriate attribution of environmental controls on DOC concentration requires that all key processes influencing variation, from episodic to seasonal to interannual time scales, are realistically represented, integrated, and shown to be applicable across a range of environmental settings. A robust, holistic, parsimonious, and transferable representation of DOC dynamics has thus remained elusive but is urgently needed, not only to constrain the land surface components of Earth system models and develop clearer insights into potential climate change feedbacks (*2*) but also to improve the understanding of the impacts of environmental change on aquatic biodiversity and drinking water supply.

The organic substances contributing to DOC in surface waters have various origins and range widely in molecular size and chemical characteristics (*17*). However, the dissolved organic matter (OM) most associated with DOC increases, sometimes referred to as “humic substances,” is characterized by colored, hydrophobic, high–molecular weight compounds indicative of terrestrial (i.e., allochthonous) sources. Isotopic analysis of this type of DOC normally indicates very recent provenance (terrestrial primary production) and generation (via OM decomposition) close to the soil surface, even in waters draining peatlands, provided that these are relatively undisturbed (*18*, *19*). Microbial decomposition, a seasonally varying temperature-dependent process (*20*, *21*), renders OM available for leaching, while the rate of OM mobilization as DOC, as well as its export to surface waters, is influenced by the chemical characteristics of leachate, which determine OM solubility, and hydrological pathways (*22*, *23*) dependent on topographic, soil, and geological properties and both current and antecedent precipitation. In organomineral soils, a notable amount of DOC may percolate into mineral layers where it becomes subject to adsorption/desorption processes and molecular transformation (*24*, *25*).

We developed a simple mathematical simulation approach to test the extent to which it is possible to explain episodic, seasonal, and long-term variation in DOC in long-term headwater records on the basis of just three linked processes: (i) thermal control of terrestrial OM decomposition rates, (ii) the effect of atmospheric deposition on soil OM solubility, and (iii) the influence of hydrological variation on DOC transport from soils to waters. Of these processes, the mechanism(s) linking deposition to solubility have arguably remained the least resolved to date and thus provide the main focus of the current study.

Rates of change in DOC concentration in long-term hydrochemical records tend to correlate negatively with rates of change in indicators of acid deposition, e.g., sulfate and chloride concentration in surface waters (*6*) and bulk deposition (*15*), with effects most acute for waters with lower base cation concentrations (*4*). The solubility of humic substances is known to be linked to the extent of complexation with protons and metal ions (*26*, *27*), and the correlative observations therefore suggest that terrestrial OM solubility is increasing in response to either, or both, a reduction in soil acidity and/or soil water ionic strength (IS), hypotheses also supported by laboratory and plot-scale manipulation experiments on DOC concentrations in the organic horizons of a range of soils (*28*–*31*).

Nevertheless, surface waters recovering from acidification do not always show correlations between DOC and water pH. For example, substantial increases in DOC were reported in two upland streams in the Czech Republic in the absence of any clear change in streamwater pH (*32*), although the increases were strongly, and inversely, correlated with streamwater IS. Similar observations were made for a range of headwater streams in the Adirondack region of the United States (*33*). Both studies concluded that reductions in soil water IS were likely to be the dominant factor influencing the DOC increases, but many other more recently published analyses of long-term DOC trends continue to overlook IS as a primary control (*34*–*39*). Furthermore, the IS of runoff itself may be considered a flawed indicator of the effects of changes in atmospheric deposition on DOC because, in the examples above, measurements of DOC and IS made essentially on the same water samples cannot be considered independent. Streamwater IS can also be influenced by changes in the proportional contributions to runoff from relatively ion-rich groundwater. Hence, during wetter periods, for example, lower IS due to baseflow dilution may be accompanied by higher DOC concentrations resulting from a switch to shallower lateral flow paths. It is therefore difficult to fully separate cause-effect relationships between streamwater DOC concentration and IS from hydrological covariance, although the Czech study also demonstrated similar DOC-IS relationships within soil water (*32*), which would be independent of this possible flow-path effect.

Unfortunately, long-term records of soil water IS that are colocated with headwater monitoring stations are rare and tend to be too plot-specific for catchment-scale assessments. We therefore tested the IS-based hypothesis within our simulations by applying the electrical conductivity (EC) of locally monitored bulk deposition, a robust and easy-to-measure surrogate for IS, as an indirect predictor of changing soil water IS (Materials and Methods). The Debye-Hückel theory (*40*) implies that the natural logarithm of ion activity in dilute solutions is inversely proportional to the square root of IS. On this basis, we reasoned that if IS is the primary factor regulating soil OM solubility and fluctuations in the EC of water leaching the upper soil organic layer are dominated by the atmospheric input of ions, then for any site, (i) the natural logarithm of DOC concentration (*lnDOC*) will vary in inverse proportion to the square root of precipitation EC (*√EC*); (ii) when both precipitation EC and *lnDOC* are expressed as proportions of long-term site medians (i.e., *lnDOC_prop_* and *EC_prop_*), then *√EC_prop_* and *lnDOC_prop_* should be related by a linear function with a slope of approximately −1 and an intercept of 1; and (iii) fits will be maximized when precipitation EC has first been averaged (precipitation-weighted) over a period representing the average response time of a catchment’s soluble OM pool to change in deposition inputs. While proportional variation in precipitation EC will be an imperfect surrogate for proportional variation in the EC of water percolating through surficial soil organic layers, we considered it to be a valid predictor of longer-term changes in soil water IS and the most robust predictor of a solubility effect available to us.

We collated multidecadal hydrochemical records with varying degrees of acid sensitivity (as determined by base cation concentration) from eight long-established headwater monitoring stations to develop our DOC simulations and test the hypothesis that IS represents a common control on soil OM solubility. The sites were drawn from the Czech Republic, Sweden, Norway, and the United Kingdom, with a requirement that locally recorded daily discharge and precipitation EC measurements, and either locally recorded or modeled daily mean air temperature data were available (see the Supplementary Materials). Half of these sites, Lysina, Pluhuv Bor, Allt a’Mharcaidh, and the River Etherow showed negative relationships between discharge and streamwater EC, thus demonstrating the limitation of using the latter as a causal predictor of the deposition-driven solubility effect.

Site-specific models were fitted by first determining the (precipitation weighted-average) smoothing window for precipitation *EC_prop_* that maximized the variance in *lnDOC_prop_* explained by the relationship described above (and see Methods and Materials). We then fitted an effect of antecedent air temperature, a surrogate for soil temperature, and a two-component discharge effect representing the (nonlinear) influence of discharge on the day of sampling to explain the residual variance. The antecedent period of smoothing for air temperature, the temperature coefficient, and the two discharge coefficients were then adjusted iteratively until model fits (determined by the variance explained) were maximized. However, the coefficient and intercept for the *√EC_prop_* variable were fixed at −1 and 1, respectively, for all sites. The final model structure is presented as Eq. 3 in Materials and Methods.

## RESULTS AND DISCUSSION

### Implications of model fits for catchment process understanding

**Fig. 1.**
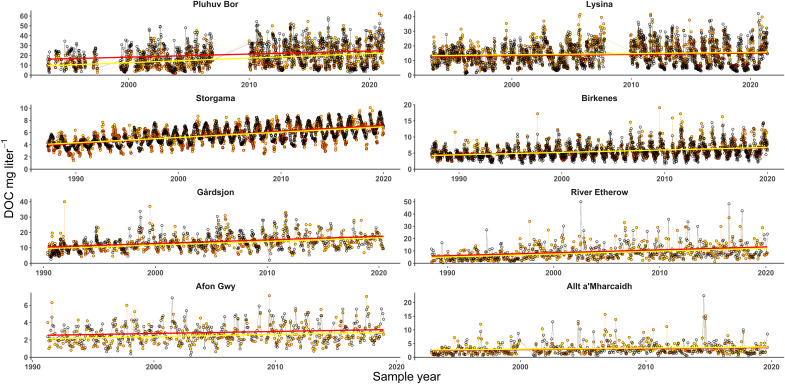
Comparison of measured (yellow) and modeled (black) DOC concentration time series in a range of long-term European headwater monitoring sites. Straight lines represent linear trends in measured (yellow) and modeled (red) DOC.

The final model fits provided highly effective simulations of DOC variation over time scales ranging from intersample to seasonal and interannual (Fig. 1), while the consistency of the final selection of antecedent smoothing windows and model coefficients across stations (Table 1) emphasized the wide-scale applicability and potential transferability of the approach. Optimal antecedent smoothing windows for temperature were significantly shorter (9 to 74 days) than those for antecedent precipitation EC (110 to 600 days). Because soil temperatures are likely to lag air temperatures by a few days, the short antecedent air temperature windows indicated that the effect of soil temperature on DOC production was relatively immediate at most sites, while the longer windows for the smoothing of the deposition EC effect indicated a more temporally smoothed influence of atmospherically deposited solutes on OM solubility.

**Table 1.** Key catchment characteristics and DOC simulation parameters. Window durations provide the number of days up to and including the day of collection of each DOC record over which the respective variables were averaged (or, in the case of precipitation EC, volume-weighted). *Q*_10_ values represent the proportional response in DOC relative to a 10°C rise in local air temperature [determined as the exponent of the temperature coefficient (α) × 10]. β_A_ and β_B_ represent nonlinear discharge effect parameters. The “discharge units and transformation” column provides the units of the original daily discharge measurements, and any multiplication factor or log transformation applied before modeling (see Materials and Methods). NA, not applicable.

Site	Catchment area (km^2^)	Runoff (m)	Catchment % peat + peaty gley cover	DOC median (mg/liter)	Precipitation EC window (days)	Air temperature window (days)	*Q*_10_	Discharge units and transformation	β _A_	β _B_	Model *R*^2^
**Storgama**	0.60	0.95	0	5.5	160	60	1.25	NA	NA	NA	0.48
**Gårdsjon**	0.04	0.52	8	11.6	169	18	1.57	ln(liter s^−1^ × 10^3^)	4.10	0.92	0.46
**Birkenes**	0.40	1.10	2	4.9	139	11	1.52	ln(liter s^−1^ × 10^3^)	0.80	0.50	0.67
**Afon Gwy**	2.10	2.14	40	2.3	110	9	1.82	ln(m^3^ s^−1^ × 10^3^)	4.65	1.08	0.50
**Allt a’Mharcaidh**	9.98	0.77	54	2.5	600	23	2.69	m^3^ s^−1^	4.40	0.85	0.47
**River Etherow**	13.0	1.09	90	6.8	270	16	2.23	m^3^ s^−1^	1.60	0.83	0.31
**Pluhuv Bor**	0.22	0.23	2	14.4	250	60	1.35	Liter s^−1^	0.16	0.64	0.78
**Lysina**	0.27	0.43	6	14.0	410	74	1.28	Liter s^−1^	0.78	0.50	0.81

Air temperature coefficients conformed with the range of incubation temperature quotients often associated with OM decomposition (*Q*_10_ range = 1.3 to 2.7) (*41*, *42*) and were positively correlated with the proportion of the catchment covered by peat and peaty gley soils, i.e., the main predominantly anoxic organic soil classes (*R*^2^ = 0.65, *P* = 0.01). With the exception of Storgama, all sites showed similar positive and curvilinear relationships between DOC and either discharge or the logarithm of discharge. DOC was most sensitive to changing discharge in the lower flow ranges and leveled out at higher flows. The absence of a detectable hydrological influence at Storgama is likely to reflect the catchment’s thin and sparsely distributed soil cover (*43*).

**Fig. 2.**
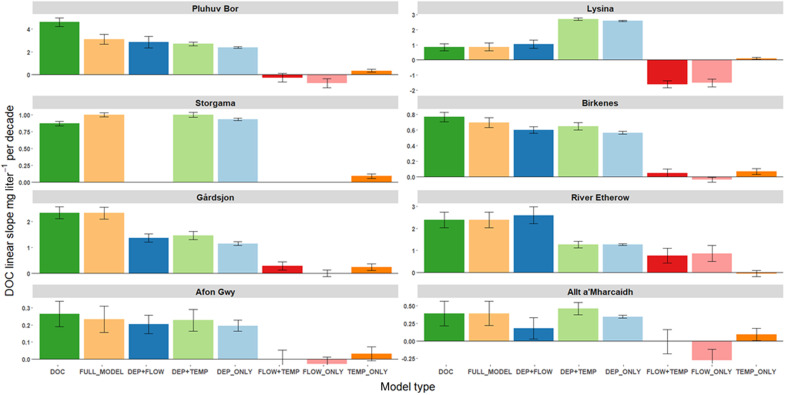
Long-term linear rates of change in DOC concentration (dark green bars), compared with rates of change in modeled DOC (light orange bars), and counterfactual alternatives (other colored bars) where the effect of one or two variables in the model has been held constant. DOC, measured DOC; FULL_MODEL, DOC modeled using all parameters; DEP, FLOW, and TEMP refer to the inclusion of deposition, discharge, and temperature as dynamic variables in the counterfactual models. Error bars represent the SE of the linear trend. Discharge is not a significant predictor of DOC in Storgama, and its effects are therefore ignored.

The models explained between 31 and 81% (mean = 56%) of the overall variance in DOC, while the slopes of the linear temporal trends in modeled DOC ranged between 67 and 115% (mean = 97%) of those of the observed DOC. The poorest model fit was achieved for the River Etherow where, unlike the other sites, discharge data were only available for a station several kilometers downstream (see the Supplementary Materials). Most variance was explained for sites where variation in DOC was dominated by either discharge (highly episodic) or temperature (strongly seasonal), but precipitation EC was clearly most important in explaining the linear trend (Fig. 2) despite the coefficient and intercept for this variable being fixed across all sites. Precipitation EC was the only variable capable of independently explaining most of the overall modeled DOC concentration slope at any site. This therefore provides strong evidence that the electrolyte solubility effect, as described by Debye-Hückel theory, represents a fundamental and universal control on terrestrial OM dynamics.

Agreement between observed and fitted DOC trends was optimized by the inclusion of one or both of the other variables, thus demonstrating their importance as either synergistic or antagonistic factors, but in the absence of a long-term reduction in precipitation EC, neither temperature nor discharge (separately or in combination) were capable of accounting for any more than a small fraction of the observed long-term DOC increase (Fig. 2). Furthermore, the model implied that the recent decline in discharge at Lysina would have resulted in an overall reduction in DOC over the past three decades had the atmospheric ion flux not fallen considerably over the same period.

**Fig. 3.**
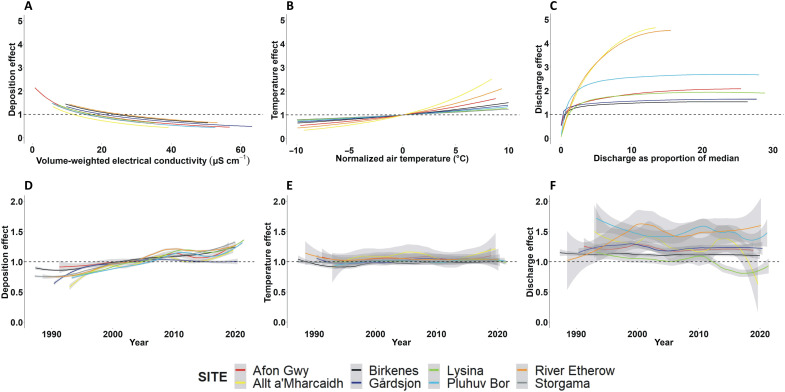
Comparison of effect sizes of the three explanatory variables. Panels represent proportional change in DOC concentration (relative to site medians), with respect to the overall range of the variables (**A** to **C**) and changes over time (**D** to **F**). Trends in temporal change are represented by loess smoothers (span = 0.33).

The discrete influence of the three variables on modeled DOC concentration across the full variable range for each site is provided in Fig. 3 (A to C), while the influence each variable exerted on modeled DOC concentrations over the monitoring period is shown in Fig. 3 (D to F). The effect of precipitation EC on overall variance in DOC concentration is of similar magnitude to the effects of temperature and discharge, but Fig. 3 (D to F) shows EC to be the only variable undergoing sufficient monotonic change at any site to account for the long-term changes observed in DOC. The figure also contrasts the relative between-site consistency of the precipitation EC and temperature effects with the more variable hydrological effect, emphasizing the importance of site-specific influences of soil structure and distribution in influencing DOC flow routing and retention.

### Implications for future DOC change

The inverse logarithmic relationship described by the Debye-Hückel equation, and at the center of our model, is very important with respect to recent and future DOC behavior. Under clean air policies across the European region and parts of North America, deposition of anthropogenic sulfur has fallen to levels not experienced since the early phases of the industrial revolution. Given the previously acknowledged links between acid deposition and DOC concentration, it might be assumed that DOC trends would mirror the recent flattening of the sulfur deposition trend, but increases in DOC continue to be reported (*6*), and it has been proposed that a more gradual reduction in the release of legacy pollutants from catchment soils, including anthropogenic sulfur, observed in some long-term hydrochemical records might account for the current apparent discrepancy (*33*).

It is possible that legacy effects could account for some of the variance in DOC not captured by our models, but the overall quality of the fits and their ability to account for the long-term DOC trend suggest that contemporary deposition remains the overriding factor, with soil OM solubility becoming increasingly responsive to unit reductions in the IS of precipitation as the latter fall toward background (i.e., preindustrial) levels. The concentration of DOC should, therefore, continue to rise for as long as ion deposition continues to fall, regardless how slowly, at least until pollutant deposition rates have fully stabilized (e.g., note the recent uptick in modeled change in DOC in response to changing precipitation EC in Fig. 3D).

The rising rate of change in the solubility of OM with decreasing IS, as described by the equation, may partly account for why relationships between DOC and sulfate and chloride concentration trends in spatial correlative studies of acid-sensitive waters have been found to be more acute in waters with low base cation concentrations (*4*, *6*) because such sites are likely to be more prevalent in regions receiving more dilute rainfall. However, it is also likely that the IS of water leaching soil organic layers with a naturally higher base cation content will be slightly less responsive to fluctuations in precipitation EC than the soils of most catchments included in this study, and this could lead to an overestimation of the effect of changing deposition IS on DOC concentrations in these cases. In formerly atmospherically contaminated coastal areas, including much of the western United Kingdom and southern and western Scandinavia, sea salt deposition is increasingly dominating the IS of precipitation as pollutant deposition declines (*44*). The relationship would suggest that the solubility of OM reaching upland drinking water supplies in these regions is becoming more sensitive to oscillations in soil water IS driven by fluctuations in storminess that govern sea salt inputs (*43*, *45*).

The influence of hydrology on DOC has long been recognized, and a component of recent DOC concentration trends has been attributed to increases in summer precipitation in parts of northern Europe (*6*, *16*). Hydrology is considered to influence DOC concentrations primarily by altering flow paths, with transport of organic compounds to waters rising as runoff is increasingly routed through the most organically rich surficial horizons. The relationships between discharge and DOC represented in our simulations provide a slightly different perspective on the role of hydrology. Model fits indicate that the DOC response to increasing runoff is most acute at low discharges and that, for most sites in our study, the effect rapidly subsides as flows increase further (Fig. 3). This is perhaps more consistent, therefore, with the effect of a reduction in the proportion of dissolved OM sequestered by mineral adsorption (*24*, *25*) as the rate of drainage increases. Consequently, the widely observed tendency for DOC to rise rapidly in the early phase of storms, a feature normally attributed to the accompanying shift in flow paths, may be partly a function of the accompanying dilution of soil water IS driving an increase in OM solubility. The resulting dilution of soil water may also help explain why DOC is sometimes reported to remain high for a period after discharge has subsided to generate an apparent hysteretic effect (*46*).

The evidence for a positive thermal effect on DOC concentration is expected, given its widely reported seasonality (*47*, *48*) and observations of slight lags with air and soil temperature (*42*). However, interannual variation in air temperatures and even long-term increases projected under climate change simulations are small relative to the amplitude of the seasonal temperature cycle, and it can be difficult to detect thermal effects on DOC beyond those that can be represented by a regular annual oscillation. Despite this, we found that when the smoothed measured air temperature in the models was replaced by a sinusoidal cycle (modeled on the same temperature data), the variance explained fell by between 1 to 2%. The size of the temperature coefficients in our models implies *Q*_10_ temperature quotients (i.e., proportional change in DOC concentration per 10°C change in smoothed air temperature) of between 1.3 and 2.7. On the assumption that soil properties, including moisture content, remain largely unchanged, we applied the relationship between temperature constants and peat and peaty gley soil cover referred to earlier to predict the impact of future regional warming on DOC concentrations in the absence of further change in other drivers. Projected increases in European air temperatures of between 2.5° and 4.0°C by the end of the current century would be expected to drive DOC increases of between 6 to 11% for an organomineral soil-dominated catchment with a *Q*_10_ of 1.3, to 28 to 49% for a peat-dominated catchment with a *Q*_10_ of 2.7.

### Implications for the spatial distribution of recent DOC change

**Fig. 4.**
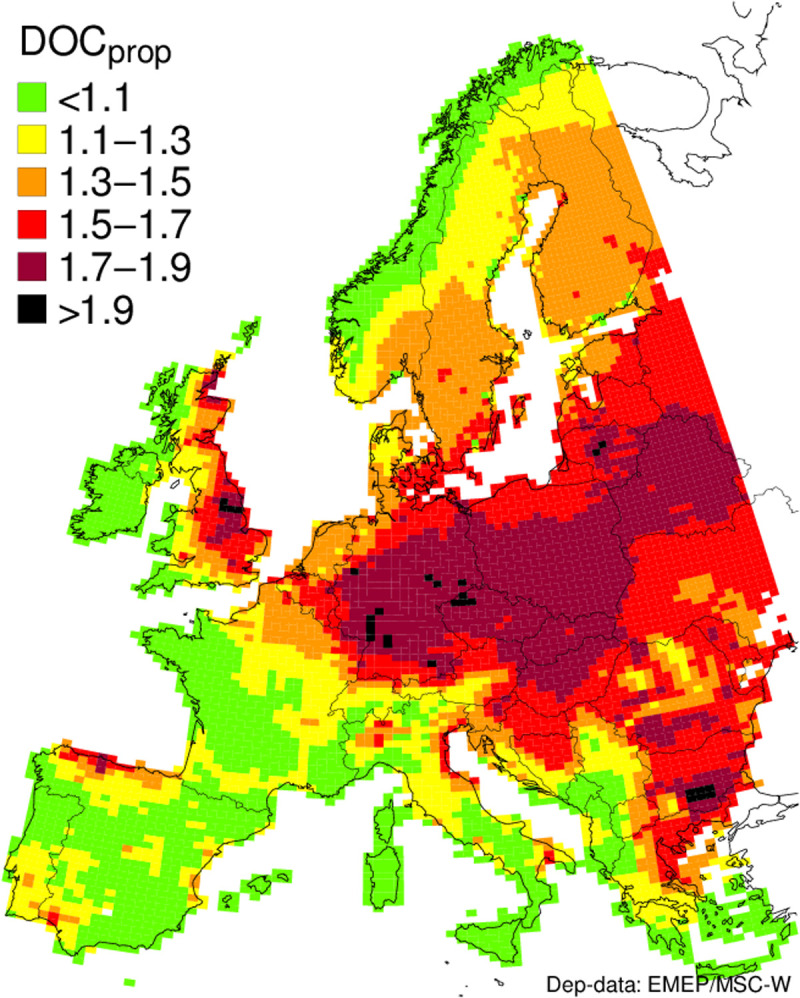
Map of the estimated effect of change in the EC of precipitation (modeled using the European Critical Loads database) on proportional change in soil water DOC concentration (*DOC_prop_*) between 1990 and 2015, according to the relationship between EC and DOC applied in the site-specific DOC simulations. Hydrological and temperature effects are not included.

On the basis of the support that our simulations provide for the importance of a deposition-dependent solubility control, we went on to model European-scale spatial variability in rates of change in soil OM solubility over the recent decades of clean air implementation using precipitation chemistry data drawn from the European Critical Loads dataset (*49*). The dataset provides estimates of change in deposition chemistry across a 0.50° × 0.25° grid. Computations of change in deposition EC and the inferred proportional change in DOC concentration (*[DOC]_prop_*) for the period 1980 to 2015 were made for about 3 million sites, west of 32°E, for forest and seminatural vegetation covering about 2.3 million km^2^ (Fig. 4; see the Supplementary Materials).

Despite the simplicity of the approach, the magnitude of modeled changes and their geographical distribution is highly consistent with observed rates of change in DOC concentration in European headwaters over similar time frames (*4*, *6*, *50*, *51*). Currently, the effects of hydrological and thermal change on spatial variation in DOC trends variations are not incorporated, resulting, for example, in a potential overestimation of DOC increases in the region of the Czech study sites, where a reduction in discharge has partially counteracted the effect of the precipitation IS decline. Nevertheless, our findings provide the foundation for the development of spatial predictions both past and future, integrating the effects of all three drivers, given the availability of the necessary explanatory data.

The map indicates that, between 1990 and 2015, the impacts of declining ion deposition on soil OM solubility will have been greatest in parts of central Europe and the eastern United Kingdom, where reductions in the concentration of sulfate and chloride in deposition have been particularly pronounced. The map also points to marked increases in soil OM solubility across wider areas of northern Europe, but impacts on surface waters will have been most obvious where organic soils are hydrologically connected to stream networks and where this source dominates stream DOC. Effects of deposition declines will have been least detectable where DOC is most strongly retained in mineral soils (*50*), where flow occurs largely over the soil surface (e.g., mire systems), and where urban and agricultural inputs of OM, or autochthonous DOC sources, dominate.

The modeling presented in this study therefore supports a holistic view of a set of interacting drivers, in which a thermally controlled pool of soluble OM is mobilized at a rate dependent on the IS of soil water, with the amount eventually exported to surface water dependent on flow routing and the rate of retention by any underlying mineral soil. The size of the soil organic pools may vary not only seasonally but also as a consequence of longer-term changes in soil management. For example, measures that change rates of OM delivery to the soil, e.g., by converting land from grassland to forestry (*52*) or changing the depth of soil aeration through drainage (*53*), might also be expected to influence DOC concentrations over time. Further insights may now also be gleaned into mineralization and sequestration of DOC within lakes and reservoirs, effects of various land management practices such as drainage, prescribed burning and grazing, and extreme events that are increasing in frequency and severity such as drought and wildfire.

With the significance of DOC fluxes and regional DOC increases in relation to the global carbon cycle now firmly recognized (*1*), there is an ongoing need for simple, generalizable models that can predict the response of these fluxes to global environmental change. Our study provides a basis to model the interacting influence of terrestrial OM decomposition, solubility, and transport of DOC export to the aquatic continuum and should enable improved prediction of the future response of this important carbon flux to changes in climate, land use, and the wider global carbon cycle.

## MATERIALS AND METHODS

### Modeling rationale

We set out to develop the most parsimonious mathematical model capable of capturing the main modes of variation in DOC concentrations in upland headwaters, from episodic to seasonal and multidecadal time scales. In particular, we were interested in testing the theory that DOC concentrations have been rising in many regions as a direct consequence of reductions in soil water IS (*32*, *33*), in turn driven by reductions in air pollutant deposition. To avoid overattributing, or misattributing, effects that could be due to other environmental influences, it was also necessary to represent other recognized drivers of terrestrial OM decomposition and land-to-river transport of DOC within the holistic model structure.

We assumed that soil temperature would govern the rate of microbial decomposition of recent primary production in the upper soil layers to create soluble OM and that fluctuations in air temperature would provide a robust surrogate for soil temperature variation, particularly when averaged over multiple days. We also reasoned that the proportion of the DOC mobilized in the soil water that is ultimately transported to the surface water would be dependent on hydrological conditions at the time of sampling, which in turn could be represented by instantaneous stream discharge.

Our primary focus, however, was to test whether the Debye-Hückel limiting law (*40*) could be applied to represent the effect of fluctuations in soil water IS on soil OM solubility. Debye-Hückel theory suggests that the solubility of humic substances in soil solution should increase with any reduction in solute strength, as the diffuse double layer around organic colloids expands and the charge shielding that promotes coagulation is consequently reduced. To some extent, fluctuations in soil pH and soil water IS are intrinsically linked. For example, reductions in IS at electrolytic concentrations typical of boreal forest soils across realistic gradients of acid deposition can also raise soil pH (*54*), although modeling of responses suggests that some of the most important groups of strong organic acids remain in solution across broad pH ranges (*33*, *55*, *56*). Because of the paucity of long-term soil water chemistry records that could be linked to most of the headwater catchments of interest, we instead gathered records of precipitation chemistry measured in samples from local bulk collectors. While precipitation monitoring protocols have not always covered the full range of ions necessary to compute IS, the monitoring of the specific EC of precipitation, which correlates very tightly with IS, is routine and analytically robust and therefore provides the best opportunity for assessing evidence for a direct effect of deposition IS on DOC concentrations.

### Sites

We identified eight long-term headwater stream monitoring stations drawn from national or regional monitoring programs in the Czech Republic, Norway, Sweden, and the United Kingdom, for which frequent (i.e., at least monthly) DOC measurements were available, together with local records for daily stream discharge, bulk precipitation EC (at least monthly), and either measured or modeled (i.e., 1-km resolution) daily air temperature, covering a period of at least 25 years (table S1).

### Stream water and bulk precipitation chemical analysis

Streamwater samples were filtered through 0.45-μm cellulose nitrate filters and analyzed for DOC using Total Organic Carbon (TOC) analyzers by first acidifying samples to purge inorganic carbon, followed by high-temperature combustion and spectrometric analysis of the resulting CO_2_. Temperature-standardized (either 20° or 25°C) EC of precipitation samples was measured at nearby precipitation chemistry monitoring stations.

### Meteorological data

Daily mean air temperature records were obtained either from local meteorological stations or, in the case of the U.K. sites, using daily 1-km resolution interpolated data provided by the Climate Hydrology and Ecology research Support System (CHESS) meteorology dataset for Great Britain (*57*). The amount of precipitation collected for each precipitation chemistry sample was used to determine precipitation-weighted EC estimates.

### Model development

DOC concentrations were modeled on a site-specific basis using a common iterative fitting approach according to the following steps.

#### Step 1

DOC concentrations were converted to proportions of site median concentrations (*[DOC]_prop_*) and then natural log-transformed (*ln[DOC]_prop_*) to achieve approximately normal distributions. Debye-Hückel theory implies that the natural logarithm of ion activity in dilute solutions is inversely proportional to the square root of IS. We assumed as our starting point, therefore, that providing the average charge density of the organic compounds had not changed significantly over time; change in *ln[DOC]_prop_* would be related to change in precipitation EC when also expressed in proportional terms (i.e., *EC_prop_*) as follows

$$ln{\left[DOC\right]}_{prop}=1-\sqrt{{EC}_{prop}}$$ (1)

where *EC_prop_* represents precipitation EC volume-weighted over a site-specific antecedent period.

For each site, we initially determined mean precipitation EC, weighted by precipitation amount, for 365 days antecedent to the collection of each DOC measurement and divided this by the median of all annual precipitation-weighted precipitation values to provide *EC_prop_*. We then applied Eq. 1 and varied the antecedent smoothing period in 10-day increments until we maximized the variance in *ln[DOC]_prop_* explained.

#### Step 2

Daily mean air temperatures for each site were normalized by subtracting the long-term mean. The seasonal pattern in the *ln[DOC]_prop_* residuals from Eq. 1, assuming this to represent the effect of temperature-dependent decomposition on soluble OM production, was modeled using sine and cosine functions applied to the day of the year. We then determined the number of days (before the day of each DOC sample) that daily mean air temperature values would need to be averaged over for the annual peaks in the smoothed air temperature records and the modeled seasonal cycle of Eq. 1 residuals to align. Regression of the *ln[DOC]_prop_* residuals against the smoothed normalized air temperature data provided an initial temperature coefficient.

#### Step 3

Exploratory data analysis had shown that while DOC appeared to vary logarithmically with both precipitation EC and temperature, relationships with discharge were either relatively linear, or DOC varied with the logarithm of discharge. We therefore next determined the exponents of the residuals from the step 2 model (i.e., EC + temperature) before plotting them against instantaneous discharge or the logarithm of discharge. In the case of the Storgama site, we observed no obvious relationship between the step 2 model residuals and discharge and therefore did not include a discharge variable in the final Storgama DOC model.

For the remaining seven sites, we observed asymptotic relationships, with the step 2 model residuals not only increasing but also leveling off at moderate to high discharges. These relationships were most effectively described by the following function

$$\text{Discharge \textbackslash{};effect}\;(\beta )=\frac{1}{\frac{\beta_{A}}{Q}-\beta_{B}+1}$$ (2)

where β*_A_* and β*_B_* are constants and *Q* is either the instantaneous discharge or the natural logarithm of instantaneous discharge, depending on the site. While not necessary for the datasets used in our study, it would be necessary to add a small constant to *Q* before log transformation should the *Q* range include values of 1 or less (regardless of units) to ensure that all transformed values are positive. Initial values for β_A_ and β_B_ were established using the Microsoft Excel “Solver” application to explain the step 2 model residuals.

#### Step 4

Because the modeled IS and thermal effects were logarithmic, the full simulation model took the following form

$${\left[DOC\right]}_{t}={\left[DOC\right]}_{\mathrm{median}}.\exp\left(1-\sqrt{\frac{{EC}_{t}}{\;{EC}_{\mathrm{median}}}}+{\alpha T}_{t}\right).\frac{1}{\frac{\beta_{A}}{Q_{t}}-\beta_{B}+1}$$ (3)

where [*DOC*]*_t_* is the DOC concentration of runoff of a site at time *t* and [*DOC*]_median_ is the long-term site median DOC concentration. Likewise, *EC_t_* represents the EC of bulk precipitation, precipitation-weighted over a site-specific period antecedent to time *t*, while *EC*_median_ represents the median of the daily computed precipitation-weighted EC*_t_* values. The term *T_t_* represents the normalized daily mean air temperature averaged over a site-specific number of days antecedent to time *t*, and the coefficient α represents the effect of the smoothed temperature variable. Last, *Q_t_* represents discharge, or the natural logarithm of discharge, depending on the site, on the day of water sampling, with the β coefficients determined according to Eq. 2.

#### Step 5

The final site-specific model calibration involved the iterative adjustment of smoothing windows for air temperature and precipitation EC, the air temperature (α), and the discharge coefficients (β*_A_* and β*_B_*) to maximize the final variance in measured [*DOC*] explained while also ensuring that the linear slopes of the modeled and observed DOC trends were in approximate agreement. The final site-specific model smoothing windows and variable coefficients for each site are indicated in Table 1 (coefficient α = the natural logarithm of the temperature quotient *Q*_10_/10).

### Model structure exploration

To consider the relative contributions of the three explanatory variables to the observed long-term DOC trends at the study sites (as presented in Figs. 2 and 3), we simply nullified the influence of one or two of the variables within Eq. 3, such that the term $\$\sqrt{\frac{{EC}_{t}}{\;{EC}_{\mathrm{median}}}}\$$ was replaced with 1, while the discharge and temperature terms were removed, as appropriate. Rates of change (with time) in observed DOC, modeled DOC according to Eq.3, and modeled DOC with one or two variables omitted, were determined by linear regression.

### Spatial modeling of the effect of the changing EC of precipitation

To consider the implications of our field observations for change in soil OM solubility at a continental scale, we applied the European Background Database (EU-DB) (*49*) to determine regional-scale change in bulk precipitation EC over the period for which data were available (i.e., 1990–2015). Computations of precipitation EC and the inferred proportional change in DOC (*DOC_prop_*) in runoff were made for about 3 million sites in Europe, west of 32°E, for forest and seminatural vegetation (EUNIS classes D through G), covering about 2.3 million km^2^.

Bulk precipitation EC (in μS/cm) was computed from the concentrations of the ions present as

$$EC=\sum_{i=1}^{n}\Lambda_{i}^{0}[X_{i}]$$ (4)

where [*X_i_*] is the concentration of ion *i* (in eq/m^3^), Λ^0^*_i_* is the (limiting) equivalent conductivity of ion *i* (in Scm^2^/eq), and *n* the number of ions.

We considered the following ions (charges suppressed): base cations, i.e.,

[BC]=[Ca]+[Mg]+[K]+[Na] (5)

Note that gradual long-term reductions in base cation deposition are known to have occurred, but neither long-term records nor modeled estimates of change were available for the European region. Consequently, base cation deposition for the year 2000 was derived from modeling conducted by van Loon *et al.* (*58*) and held constant for the full time series and the “pollutants” sulfate, [SO_4_], nitrate [NO_3_] and ammonium [NH_4_], and chloride [Cl].

The bicarbonate concentration [HCO_3_] was computed from the proton concentration, [*H*], as

$$\left[{\mathrm{HCO}}_{3}]=K_{{\mathrm{HCO}}_{3}}/[H\right]$$ (6)

where *K*_HCO3_ is the equilibrium constant. [H] itself was then computed from the charge balance

$$[H]-[{\mathrm{HCO}}_{3}]+[\mathrm{ANC}]=0$$ (7)

with the ANC (acid-neutralizing capacity) defined as

$$\left[\mathrm{ANC}]=[\mathrm{BC}]-[\mathrm{Cl}]-[{\mathrm{SO}}_{4}]-[{\mathrm{NO}}_{3}]+[{\mathrm{NH}}_{4}\right]$$ (8)

Equation 7 becomes a quadratic equation in [*H*] after inserting Eq. 6, and the (only positive) solution is

$$[H]=\frac{1}{2}\left(\sqrt{{\left[\mathrm{ANC}\right]}^{2}+4K_{{\mathrm{HCO}}_{3}}}-[\mathrm{ANC}]\right)$$ (9)

We define the following property, *DOC_prop_*, as

$${DOC}_{prop}=\exp\left(1-\sqrt{{EC}_{2015}/{EC}_{1990}}\right)$$ (10)

where EC_1990_ and EC_2015_ are the conductivities computed with N- and S-depositions from the years 1990 and 2015, respectively.

Using data from the European Background Database, the concentrations of ion *X* were computed from its wet deposition flux, *X*_wet_, as follows

$$[X]=X_{\mathrm{wet}}/P$$ (11)

where *P* is the annual precipitation.

Figure S1 presents the grid average wet deposition ratio used in the calculations. Figure S2 shows the cumulative distribution functions (cdfs) of EC (see Eq. 4) for two deposition years (1990 and 2015) for all sites west of 32°E (about 3 million sites). It also shows that, for example, the 90th percentile in 2015 is around 20 μS cm^−1^ in 2015 and above 80 μS cm^−1^ in 1990. Median EC values are mapped in fig. S3. Figure S4 provides the cdfs of DOC_prop_ for all sites, while the spatial distribution of the *DOC_prop_* values (see Eq. 10) of all sites can be seen in the maps in fig. S5.
