# Supplementary material for: Long-term rise in riverine dissolved organic carbon concentration is predicted by electrolyte solubility theory
Source: Sci Adv. 2023 Jan 18;9(3):eade3491. doi: 10.1126/sciadv.ade3491 (PMC9848639; doi:10.1126/sciadv.ade3491)
Supplement: Supplementary file 1 — Figs. S1 to S6 Table S1 [file sciadv.ade3491_sm.pdf]

Science Advances
AAAS

Supplementary Materials for

Long-term rise in riverine dissolved organic carbon concentration is predicted by electrolyte solubility theory

Donald T. Monteith *et al.*

Corresponding author: Donald T. Monteith, donm@ceh.ac.uk

Sci. Adv. **9**, eade3491 (2023)
DOI: 10.1126/sciadv.ade3491

This PDF file includes:

Figs. S1 to S6
Table S1

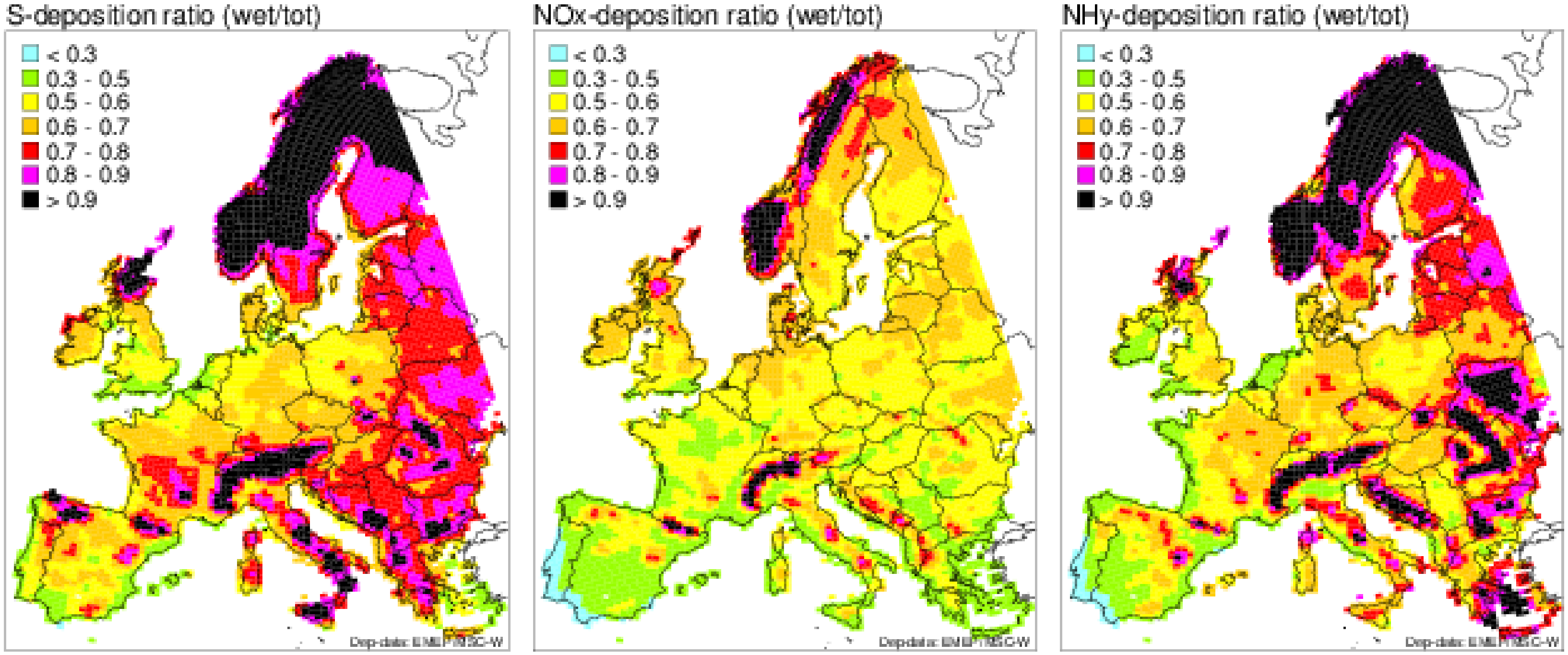

Fig. S1. Modelled fractions of wet and dry sulphur and nitrogen species. Average wet deposition fraction, i.e. ratio of wet over total deposition in each 0.50°×0.25° grid cell west of 32°E for S (left), NO_x (centre) and NH_y (right).

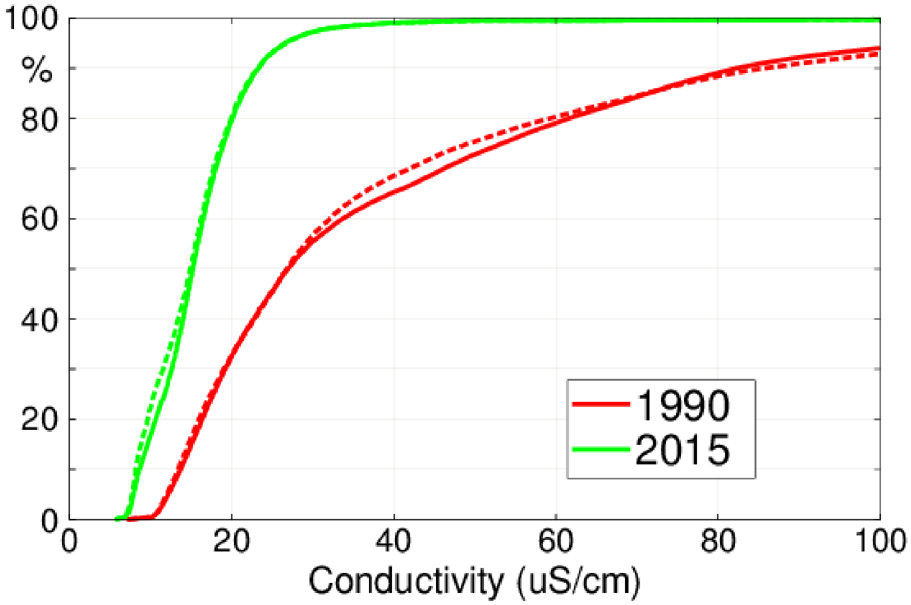

Fig. S2. Cumulative distribution functions (cdfs) of modelled precipitation electrical conductivity across Europe. Cdf of electrical conductivity (EC) in Europe west of 32°E (about 3 million sites) for deposition in 1990 (red) and 2015 (green). The dashed cdfs are for sites with organic soils (about 1.2 million sites).

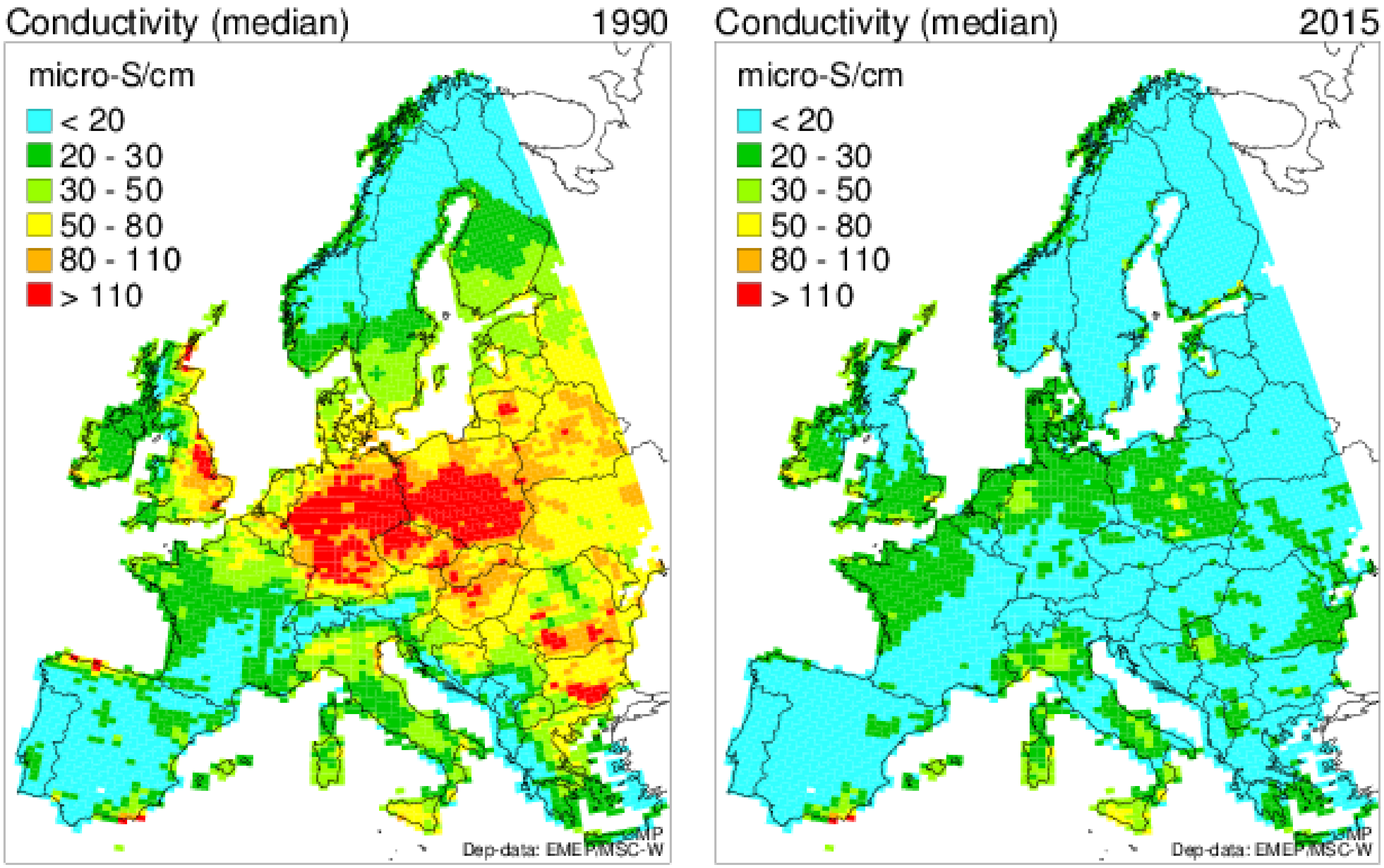

Fig. S3. Modelled precipitation electrical conductivity. Maps of median electrical conductivity (EC) (see Equation 1) for all sites in Europe for depositions in 1990 (left) and 2015 (right) on a 0.50°×0.25° grid.

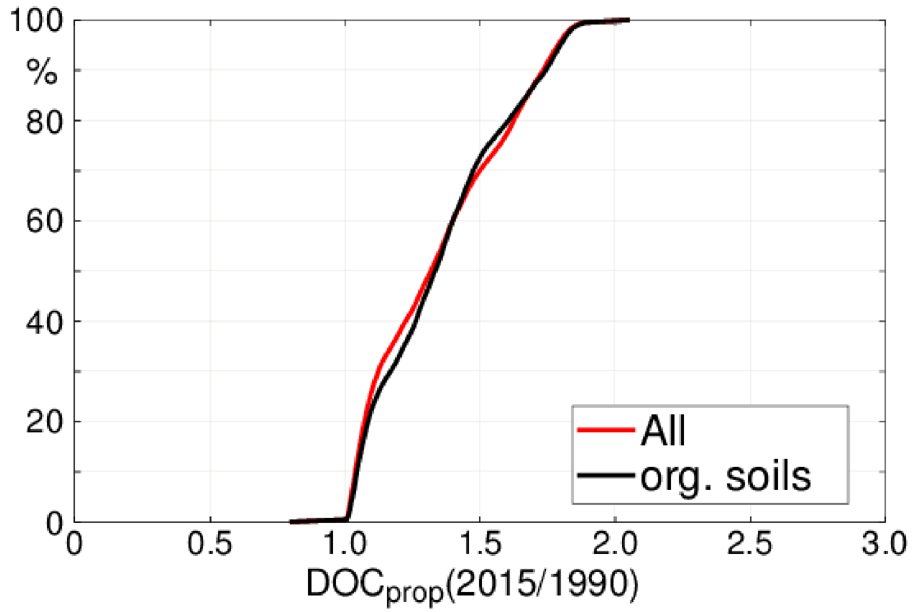

Fig. S4. Cumulative distribution functions for modelled response in soil organic matter solubility to change in precipitation electrical conductivity. Cdf of the quantity DOC_{prop} (see eq.7) using N- and S-depositions for the years 1990 and 2015 for about 3 million sites in Europe (red line) and about 1.2 million sites with organic soils (black line).

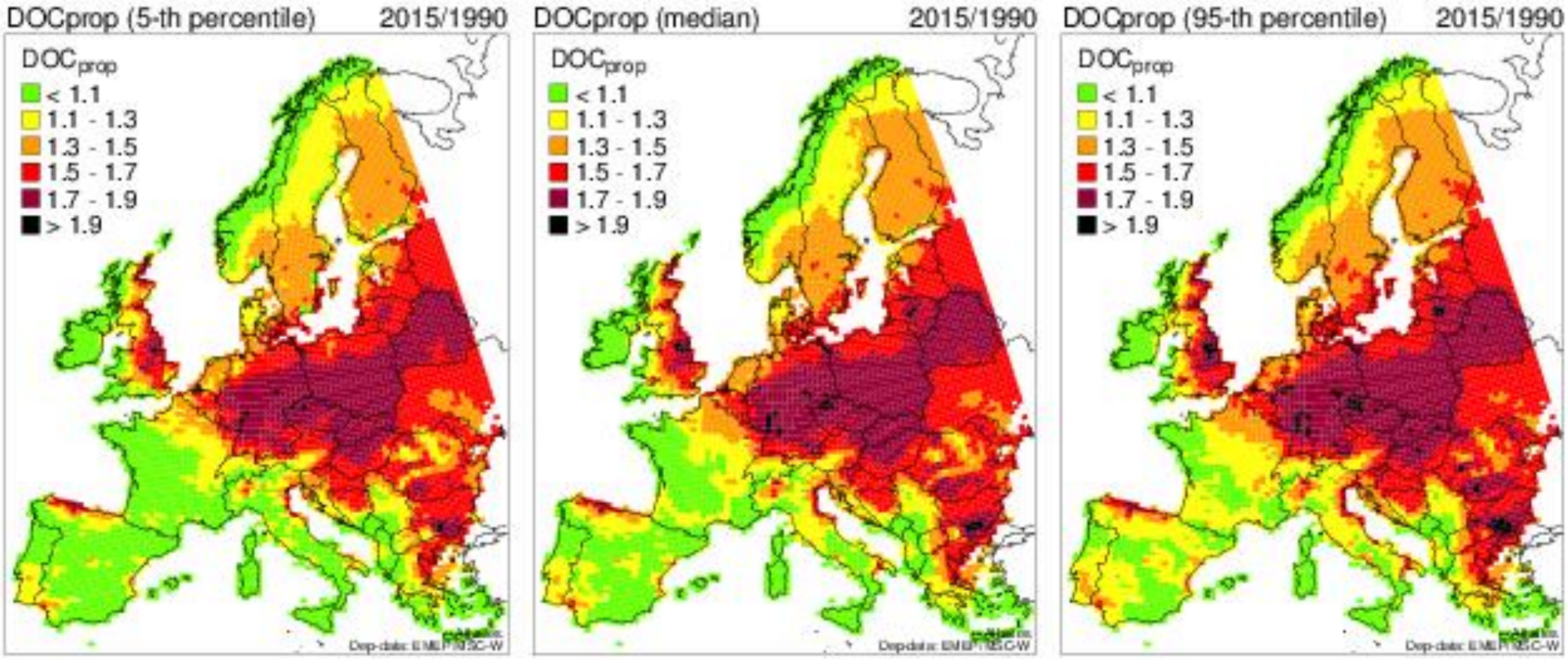

Fig. S5. Maps of modelled change in soil organic matter solubility by 2015 relative to 1990. Maps of DOC_{prop} in response to change in N and S deposition for the years 1990 and 2015 for all sites, displayed as 5-th percentile (left), median (centre) and 95-th percentile (right) in a 0.50°×0.25° grid.

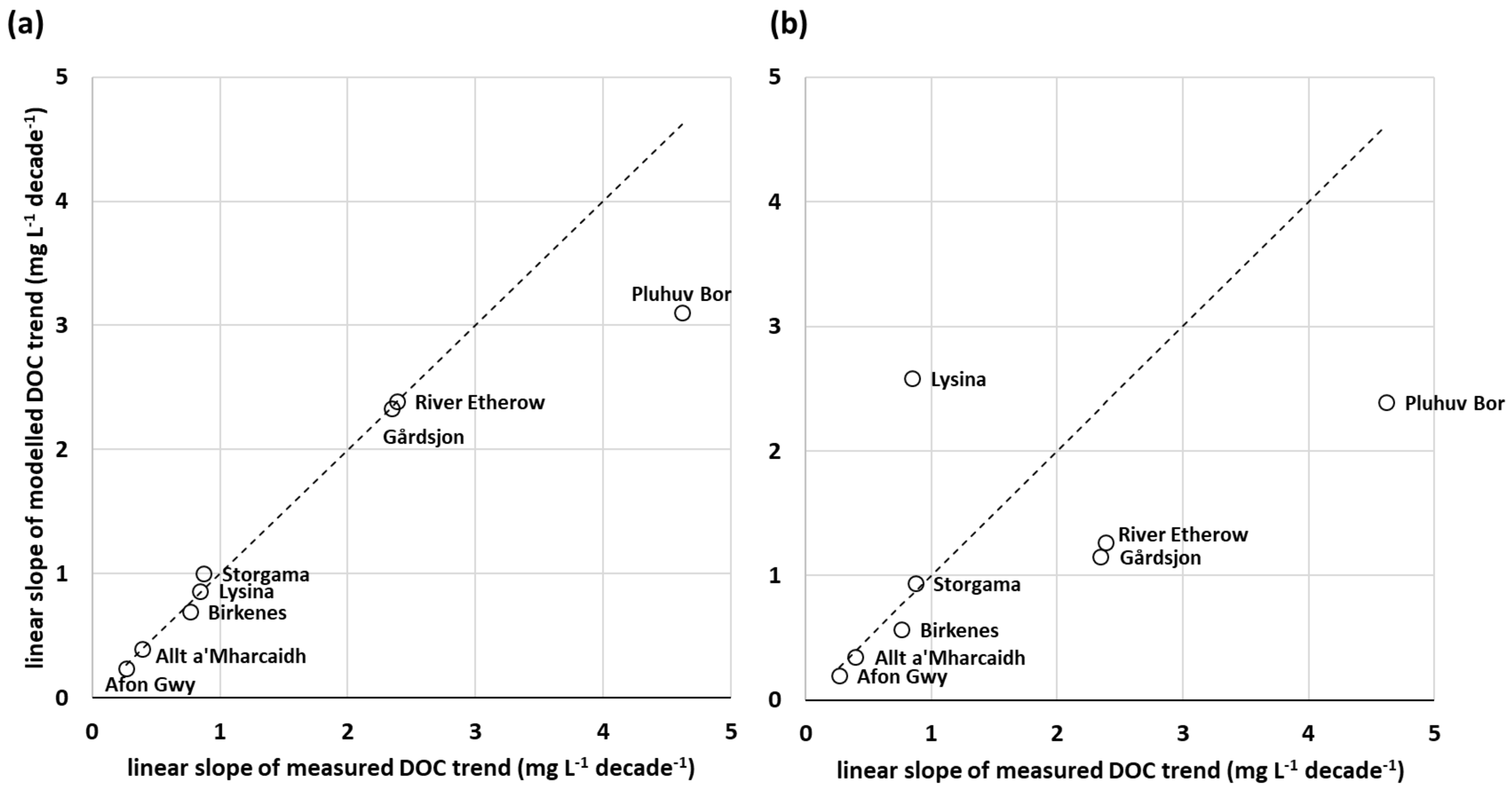

Fig. S6. Relationships between linear slopes in observed DOC concentration and slopes in (a) modelled DOC (full model) and (b) modelled DOC when effects of temperature and discharge are held constant (i.e. trends are driven by the modelled effect of changing precipitation EC only).

Table S1. Summary of monitoring site locations and data sources. UWMN = UK Upland Waters Monitoring Network; CGS = Czech Geological Survey; NIVA = Norwegian Institute for Water Research; SEPA = Swedish Environmental Protection Agency; UKCEH-CHESS = UK Climate, Hydrology and Ecology research Support System. Note: the monitoring stations used to provide discharge data for the Allt a'Mharcaidh and River Etherow are situated approximately 3 and 17 km downstream of the water sampling sites respectively.

	site	lat	lon	altitude	period	data sources			
**country**				**range (m)**		**DOC**	**daily discharge**	**precipitation chemistry**	**air temperature**
**UK**	Allt a'Mharcaidh	57.1190	-3.8514	325-998	1992-2019	UWMN	https://nrfa.ceh.ac.uk/data/station/info/8013	https://uk-air.defra.gov.uk/data/non-auto-data?network=ukeap&uka_id=UKA00086	UKCEH-CHESS
**UK**	River Etherow	53.4930	-1.8253	280-633	1988-2020	UWMN	https://nrfa.ceh.ac.uk/data/station/info/69015	https://uk-air.defra.gov.uk/data/non-auto-data?network=ukeap&uka_id=UKA00119	UKCEH-CHESS
**UK**	Afon Gwy	52.4539	-3.7320	440-730	1991-2018	UWMN	https://nrfa.ceh.ac.uk/data/station/info/55033	https://uk-air.defra.gov.uk/data/non-auto-data?network=ukeap&uka_id=UKA00173	UKCEH-CHESS
**Czech R.**	Lysina	50.0167	12.6500	829-949	1993-2021	CGS	CGS	CGS	CGS
**Czech R.**	Pluhuv Bor	50.0500	12.7662	690-804	1993-2021	CGS	CGS	CGS	CGS
**Norway**	Birkenes	58.3833	8.2500	200-300	1987-2019	NIVA	NIVA	Norwegian Institute of Air Research	NIVA
**Norway**	Storgama	60.3666	9.6500	580-690	1987-2019	NIVA	NIVA	Norwegian Institute of Air Research	NIVA
**Sweden**	Gårdsjön F1	58.0550	12.0208	113-141	1990-2020	SEPA	SEPA	SEPA	SEPA
